# Gut microbiota of frugo-folivorous sifakas across environments

**DOI:** 10.1186/s42523-021-00093-5

**Published:** 2021-05-18

**Authors:** Lydia K. Greene, Marina B. Blanco, Elodi Rambeloson, Karlis Graubics, Brian Fanelli, Rita R. Colwell, Christine M. Drea

**Affiliations:** 1 Program in Ecology, Duke University, Durham, NC 27708 USA; 2 The Duke Lemur Center, Durham, NC 27705 USA; 3 Department of Biology, Duke University, Durham, NC 27708 USA; 4 The Anjajavy Lodge and Reserve, Anjajavy, Sofia Region Madagascar; 5 CosmosID, Inc., Rockville, MD 20850 USA; 6 University of Maryland Institute of Advanced Computer Studies, University of Maryland, College Park, MD 20742 USA; 7 Department of Evolutionary Anthropology, Duke University, Durham, NC 27708 USA

**Keywords:** Amplicon sequencing, Captivity, Folivory, Gut microbiome, Husbandry, Lemur, Madagascar, Metagenomic sequencing, Strepsirrhine primate

## Abstract

**Background:**

Captive animals, compared to their wild counterparts, generally harbor imbalanced gut microbiota owing, in part, to their altered diets. This imbalance is particularly striking for folivores that fundamentally rely on gut microbiota for digestion, yet rarely receive sufficient dietary fiber in captivity. We examine the critically endangered Coquerel’s sifaka (*Propithecus coquereli*), an anatomically specialized, rather than facultative, folivore that consumes a seasonal frugo-folivorous diet in the wild, but is provisioned predominantly with seasonal foliage and orchard vegetables in captivity. Using amplicon and metagenomic sequencing applied to fecal samples collected from two wild and one captive population (each comprising multiple groups), we clarify how dietary variation underlies the perturbational effect of captivity on the structure and function of this species’ gut microbiota.

**Results:**

The gut microbiota of wild sifakas varied by study population, most notably in community evenness and in the abundance of diet-associated microbes from *Prevotellaeceae* and *Lachnospiraceae*. Nevertheless, the differences among wild subjects were minor compared to those evident between wild and captive sifakas: Unusually, the consortia of captive sifakas were the most diverse, but lacked representation of endemic *Bacteroidetes* and metagenomic capacity for essential amino-acid biosynthesis. Instead, they were enriched for complex fiber metabolizers from the *Firmicutes* phylum, for archaeal methanogens, and for several metabolic pathways putatively linked to plant fiber and secondary compound metabolism.

**Conclusions:**

The relatively minor differences in gut microbial structure and function between wild sifaka populations likely reflect regional and/or temporal environmental variability, whereas the major differences observed in captive conspecifics, including the loss of endemic microbes, but gain in low-abundance taxa, likely reflect imbalanced or unstable consortia. Indeed, community perturbation may not necessarily entail decreased community diversity. Moreover, signatures of greater fiber degradation indicate that captive sifakas consume a more fibrous diet compared to their wild counterparts. These results do not mirror those typically reported for folivores and herbivores, suggesting that the direction and strength of captivity-induced ‘dysbiosis’ may not be universal across species with similar feeding strategies. We propose that tailored, species-specific dietary interventions in captivity, aimed at better approximating naturally foraged diets, could functionally ‘rewild’ gut microbiota and facilitate successful management of diverse species.

**Supplementary Information:**

The online version contains supplementary material available at 10.1186/s42523-021-00093-5.

## Background

**Fig. 1:**
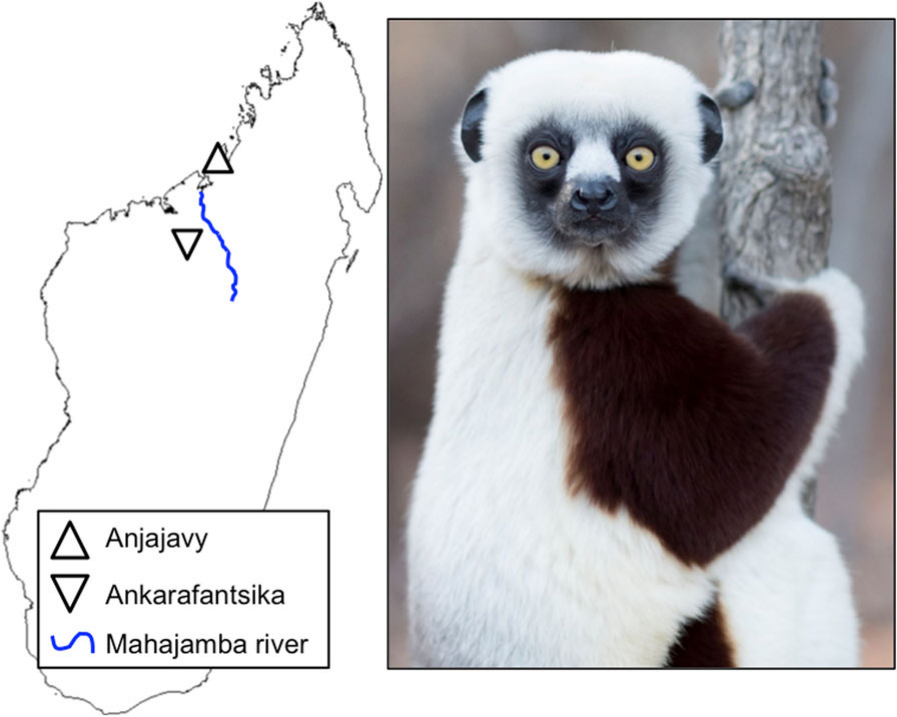
Map of Madagascar highlighting the two field sites, including the Anjajavy Protected Area (up triangle) and Ankarafantsika National Park (down triangle), separated by the Mahajamba river (blue line), and photo of the study species, the Coquerel’s sifaka (*Propithecus coquereli*). Photo by Sara Clark

The gut microbiota of animal hosts perform vital functions that support nutrition, promote health, and underlie natural host behavior [1–3]. These key roles have led to increasing calls for microbiome science to be incorporated into conservation biology and wildlife management [4–7]. Notably, a major finding of the last decade is that, compared to their wild counterparts, captive animals harbor ‘dysbiotic’ or imbalanced gut microbiota [8, 9] that may negatively influence host health [10] and inhibit reintroduction of captive animals into the wild [4, 11]. Husbandry initiatives aimed at restoring or ‘rewilding’ the gut microbiota of captive animals are thus timely and could benefit from comparisons of microbial community composition and metabolic function between multiple populations of wild and captive conspecifics. Here, using amplicon and metagenomic sequencing, respectively, we determine gut microbiome structure and function in three populations of the critically endangered Malagasy primate, the Coquerel’s sifaka (*Propithecus coquereli*). Our two sites for wild populations include the ‘Anjajavy’ Protected Area and the ‘Ankarafantsika’ National Park, Madagascar (Fig. 1); our site for the sole captive population is the Duke Lemur Center, ‘DLC,’ in North Carolina.

Captivity-induced microbial dysbiosis has called into question the validity of using captive hosts to address ecological and evolutionary questions regarding animal microbiota [12–14], while also underscoring the significant husbandry complications that derive from keeping wildlife in captivity [6]. Nonetheless, not all hosts are equally susceptible to such perturbation [9, 15, 16]: the changes in microbial community diversity, composition or taxonomic abundance can depend on the host’s phylogenetic placement and feeding strategy. Already, several mechanisms have been proposed to explain the presence and variability of captivity-induced dysbiosis [6], including housing animals in sterilized environments that minimize their exposure to microbes, prescribing frequent antibiotics and antimicrobials that disrupt microbial community dynamics, and providing diets that do not adequately replicate those naturally foraged.

The dietary mechanism of captivity-induced microbial dysbiosis has received particular attention in herbivores (or plant eaters) and folivores (or leaf eaters) [8, 10, 15, 17, 18]—species most susceptible to perturbation [15] because they fundamentally rely on gut microbial action to meet their own nutritional demands [2, 19]. Normally, gut microbes ferment recalcitrant plant fibers into nutritious short-chain fatty acids [20], metabolize plant secondary compounds to improve nutrient bioavailability [21, 22], and produce nutrients that mammals cannot endogenously synthesize, including essential vitamins and amino acids [2, 23]. Across phylogenetic lineages, the gut microbiota of herbivores and folivores broadly follow a phylosymbiotic pattern [16, 24–26], suggesting that distant relatives acquired unique microbial solutions to solve the challenges inherent to folivory. The success of these feeding strategies thus depends on dietary co-specialization between the host and its gut microbiota established over evolutionary time. Yet, within the constraints imposed by phylogenetic placement, herbivore and folivore gut microbiota are also shaped by dietary niche and local environment [27, 28], responding in real time to dietary changes across daily and seasonal timescales [29–33]. In captivity, diets typically shift from natural foliage to commercial chow, orchard vegetables, and locally available foliage and are typically associated with sizable reductions in microbial diversity, marked shifts in community composition, and a tradeoff between recalcitrant-fiber and simple-fiber metabolizers [8, 10, 17]. Captive grazers and leaf-eaters thus consume more readily digestible diets that lack sufficient recalcitrant fiber and tannins, but are enriched for simple fibers, fats, and available proteins [34–36]. In some cases, increased access to naturalized diets and/or grassy or forested enclosures can help restore gut microbial diversity and taxonomic composition [8, 10, 16, 18], indicating that the perturbational effect of captivity is, at least partially, reversible.

The Coquerel’s sifaka is one of only a few folivorous lemurs to survive in captivity, making it a unique system in which to examine natural variation in gut microbiota, as well as the perturbational effect of captivity. Sifakas have numerous anatomical adaptations to facilitate folivory and hindgut fermentation, including an elongated gastrointestinal tract and sacculated cecum [37], but they are most accurately classified as seasonal frugo-folivores: In the wild, fruits and flowers can account for ~ 5–80% of an otherwise predominately leaf-based diet [38, 39]. In captivity, however, sifakas are provisioned with local foliage, orchard vegetables, and fibrous chow. Under the hypothesis that local environmental conditions drive functional variation in gut microbiota, particularly in dietary specialists such as folivores and herbivores [27, 28], we predict that 1) the geographically distinct populations of wild sifakas will vary in their microbial communities and metagenomic profiles, and 2) the consortia of captive sifakas will be markedly distinct from those of their wild counterparts. In particular, like other folivores and herbivores [8, 10, 17], we expect captive sifakas to exhibit reduced gut microbial diversity, with significantly altered taxonomies and metabolic capacities. If dietary mechanisms specifically underlie gut microbial dysbiosis in captive animals, relative to their wild counterparts [6, 10], we expect captive sifakas, that consume less fruit, but abundant foliage, to harbor gut communities with reduced signatures of fruit and sugar metabolism, but perhaps greater signatures of fiber fermentation. Insights into the links between host feeding strategy, dietary variability, and the perturbational effect of captivity, gained from these comparisons, could inform future conservation strategies and dietary intervention for this flagship species and other dietary specialists in captivity.

## Results

### The structure of the sifaka gut microbiome across populations

**Fig. 2:**
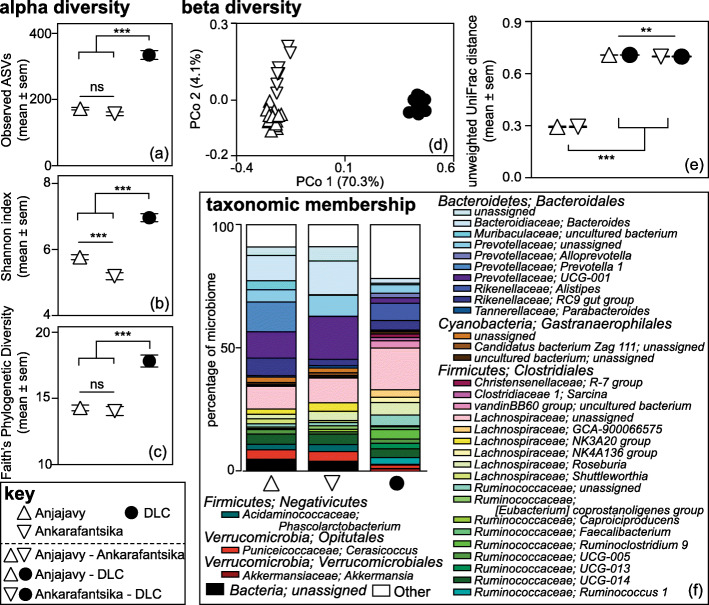
Gut microbiome structure in Coquerel’s sifakas (*Propithecus coquereli*), including wild populations living in the Anjajavy Protected Area (up triangle) and Ankarafantsika National Park (down triangle), and the captive population at the Duke Lemur Center (DLC, black circle). Depicted are results of alpha diversity, including: **a** Observed ASVs, **b** the Shannon index, and **c** Faith’s Phylogenetic Diversity; beta diversity, including unweighted UniFrac distances graphed in **d** Principal Coordinate (PCo) space and as **e** pairwise comparisons, and; **f** taxonomic membership, graphed as stacked bar charts of all the microbial genera that accounted for > 1% of the total microbiome, for which color families reflect microbial phyla and orders, and distinct shades reflect distinct species. “Other’ refers to the summation of all taxa that failed to reach 1% of the total microbiome. ** *p* < 0.01; *** *p* < 0.001; ns *p* > 0.1

Our three sifaka populations, comprised 50 animals (Table 1); the 46 that contributed to amplicon sequencing harbored structurally different gut microbiota, with those from the DLC being the most distinct (Fig. 2). Population identity was significantly associated with alpha diversity, as captured by Observed Amplicon Sequence Variants (ASVs) (ANOVA: F_2,25_ = 181.37, *p* < 0.001; Fig. 2a), the Shannon index (ANOVA: F_2,25_ = 135.070, *p* < 0.001; Fig. 2b), and Faith’s Phylogenetic Diversity (ANOVA: F_2,25_ = 57.573, *p* < 0.001; Fig. 2c). *Post-hoc,* pairwise comparisons revealed that these results were largely driven by captive sifakas. Only the Shannon index showed a significant difference between the two wild populations, with sifakas from Anjajavy harboring greater diversity than did  sifakas from Ankarafantsika (Tukey test: *p* < 0.001). Otherwise, contrary to prediction, the consortia of captive sifakas, relative to both wild populations, had significantly *greater* values across all three alpha diversity measures (Tukey tests for both comparisons per metric: *p*s < 0.001).

**Table 1:** Study subjects, sample sizes, and methods used

Sifaka population	Number of subjects	Number of social groups	Sample storage		Sequencing analyses	
			frozen	buffer	amplicon	metagenomic
Anjajavy	22	8	0	22	22	4
Ankarafantsika	9	5	0	9	9	4
Duke Lemur Center	19	9	19	15	30^a^	4

^a^Paired samples from the same defecation event split between storage conditions (see supplementary material)

Nested within each study population (Table 1) were multiple (*n* = 5–9) social groups that were significantly associated with Observed ASVs (ANOVA: F_17,25_ = 2.06, *p* = 0.049) and Shannon index (ANOVA: F_17,25_ = 2.372, *p* = 0.024), and modestly associated with Phylogenetic Diversity (ANOVA: F_17,25_ = 1.98, *p* = 0.058). These associations revealed even finer-scale heterogeneity in gut microbiota structuring within populations.

The identity of the three populations was also associated with beta diversity, as captured by ‘unweighted’ and ‘weighted’ UniFrac distances. Study population was significantly associated with both metrics (PERMANOVAs unweighted: *R*^2^ = 0.74, F_2,25_ = 82.24, *p* < 0.001; Fig. 2d; weighted: *R*^2^ = 0.71, F_2,25_ = 91.32, *p* < 0.001), respectively explaining 74 and 71% of the variation across samples. *Post-hoc* pairwise comparisons revealed that sifakas in the two wild populations significantly differed in metrics of community distance (unweighted: *R*^2^ = 0.46, *p* = 0.003; weighted: *R*^2^ = 0.34, *p* = 0.003); however, the greatest differences were consistently between wild and captive sifakas (unweighted: *R*^2^ > 0.97, *p* = 0.003 for both comparisons; weighted: *R*^2^ > 0.93, *p* = 0.003 for both comparisons).

Again, nested within study population, social group was significantly associated with these beta diversity metrics (PERMANOVAs for unweighted: *R*^2^ = 0.14, F_17,25_ = 1.86, *p* = 0.022; weighted: *R*^2^ = 0.19, F_17,25_ = 2.85, *p* = 0.001), respectively explaining an additional 14 and 19% of the variation across samples.

Pairwise comparisons of UniFrac distances between populations further highlight population-level variation. Overall, there were significant differences in mean pairwise distances between the three study populations (Kruskal-Wallis tests for unweighted: H = 408.7, *p* < 0.001; weighted: H = 387.9, *p* < 0.001), with *post-hoc* tests confirming that the distances between wild-captive pairs, from either wild population, were significantly greater than were the distances between wild-wild pairs (Dunn’s tests for both comparisons per metric: *p*s < 0.001; Fig. 2e). *Post-hoc* tests also confirmed that unweighted UniFrac distances between Anjajavy-DLC pairs were more similar than were those of Ankarafantsika-DLC pairs (Dunn’s test: *p* = 0.005), whereas tests of weighted UniFrac distances suggested that the consortia of wild-captive pairs were equally dissimilar at both sites (Dunn’s test: *p* = 0.99).

Regarding taxonomic composition, sifakas from all three populations harbored consortia dominated by taxa from the *Bacteroidetes* and *Firmicutes* phyla, and *Bacteroidales* and *Clostridiales* orders, with lesser contributions from members of the *Cyanobacteria* and *Verrucomicrobia* phyla. *Bacteroidetes* were more abundant in the consortia of wild sifakas, accounting for 53 and 49% of sequences from sifakas in Anjajavy and Ankarafantsika, respectively, but accounting for only 23% of sequences in DLC sifakas. In contrast, *Firmicutes* were more abundant in the gut microbiota of captive sifakas, accounting for 68% of sequences, but for only 29 and 33% of sequences from sifakas in Anjajavy and Ankarafantsika, respectively.

**Fig. 3:**
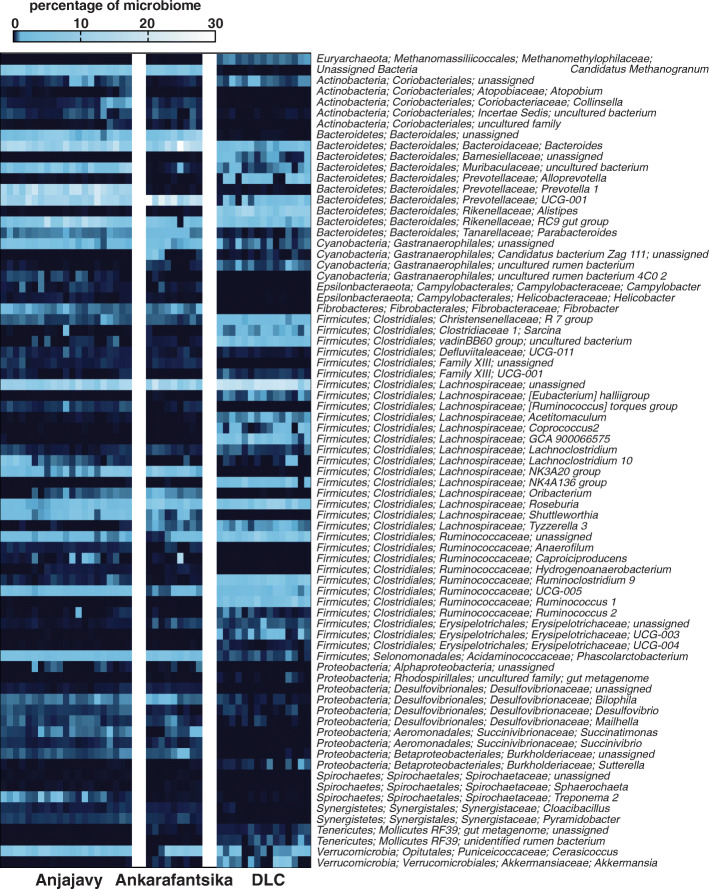
Heat map of the microbial genera that were significantly enriched in one of the three study populations, including wild sifakas living in the Anjajavy Protected Area or Ankarafantsika National Park and captive sifakas housed at the Duke Lemur Center (DLC). Rows depict the percentages of each microbe relative to the total microbiome, with abundances increasing from dark to light colors. The microbes’ phylogenetic phyla, orders, families, and genera (as available) are provided on the right. Each column represents one sample from one sifaka

Below the phylum level, there were notable differences in taxonomic profiles among the three populations, with those of captive sifakas being the most distinct (Fig. 2f). In total, Linear Discriminant Analysis Effect Size (LEfSe) identified 79 microbial genera significantly enriched in one of the three study populations, with 75 of these genera significantly enriched following additional correction for multiple testing (Fig. 3). Sifakas living in Anjajavy, compared to those in Ankarafantsika, had greater abundances of microbes like *Prevotella 1* and *Parabacteroides*; however, all wild sifakas, compared to captive sifakas, had greater abundances of *Bacteroides, Phascolarctobacterium*, and *Akkermansia,* as well as many *Prevotellaceae*. Wild sifakas had greater abundances of bacterial taxa that could not be assigned below domain-level resolution (i.e., microbes not yet present in online databases). Whereas these unassigned microbes accounted for 4.84 and 3.99% of the taxa of Anjajavy and Ankarafantsika sifakas, respectively, they accounted for only 0.007% of the taxa in captive sifakas. In contrast, captive sifakas showed greater abundance of many well-known bacterial taxa in the *Rikenellaceae, Lachnospiraceae,* and *Ruminococcaceae* families, as well as archaea from the *Euryarchaeota* phylum. Captive sifakas also had a greater number of microbes present at low relative abundances, that is, microbial genera accounting for < 1% of sequences, on average, across individuals.

### Function of the sifaka gut microbiota across populations

From a subset of 12 fecal samples (Table 1), we characterized the gut metagenome across study populations relative to whether sifakas were wild or captive. Overall, wild and captive sifakas harbored functionally distinct microbiota, as captured by differences in the presence and relative abundance of metabolic pathways. We identified 142 metabolic pathways from the MetaCyc database in the sifakas’ metagenome, 34 (24%) of which were present in only one study population. Among population-specific pathways were those for essential amino-acid biosynthesis and fermentation. For example, the microbiota of Anjajavy sifakas uniquely showed one pathway for L-phenylalanine biosynthesis (PWY-6318), whereas those of Ankarafantsika sifakas uniquely showed the mixed-acid fermentation pathway (FERMENTATION-PWY), and those of DLC sifakas uniquely showed one pathway for the production of the short-chain fatty acid butyrate from pyruvate (CENTFERM-PWY).

**Fig. 4:**
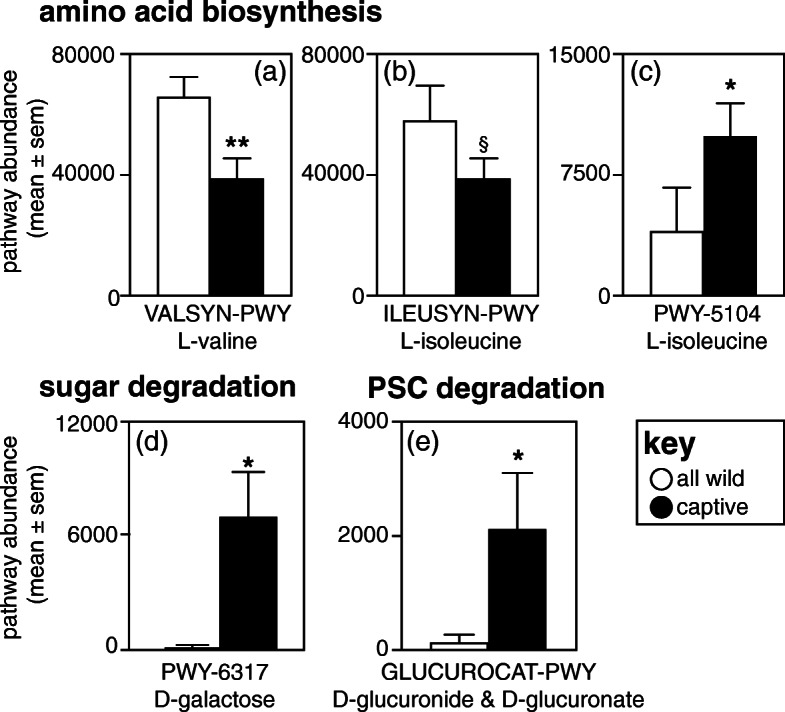
Gut microbiome function in three populations of Coquerel’s sifakas (*Propithecus coquereli*), including wild populations living in the Anjajavy Protected Area and Ankarafantsika National Park (white bars) and the captive population housed at the Duke Lemur Center (DLC, black bars). Depicted are results of key metabolic pathways that were differentially enriched in either wild or captive populations, including the biosynthesis of essential amino acids **a** L-valine and **b**, **c** L-isoleucine, and the degradation of **d** D-galactose via the Leloir pathway and of **e** D-glucuronide and D-glucuronate. The identifying PWY codes of the metabolic pathways are from the MetaCyc database. § *p* < 0.10; * *p* < 0.05; ** *p* < 0.01

From the subset of 108 pathways that were shared among populations, LEfSe analysis identified 19 that were differentially enriched between wild and captive sifakas, reflecting differences in amino acid, fatty acid, and vitamin biosynthesis, plant fiber and sugar degradation, and potential plant secondary compound metabolism (Fig. 4). In reference to amino acid metabolism, the microbiota of wild sifakas showed greater capacity for biosynthesizing L-valine (VALSYN-PWY: log (LDA) = 4.16, *p* = 0.008; Fig. 4a) and L-glutamine (PWY-5505: log (LDA) = 3.62, *p* = 0.05; Fig. 4b), and trended towards greater capacity for biosynthesizing L-isoleucine from threonine (ILEUSYN-PWY: log (LDA) = 4.13, *p* = 0.06; Fig. 4b). In contrast, the microbiota of captive sifakas showed greater capacity for biosynthesizing L-isoleucine from other precursors (PWY-5104: log (LDA) = 3.62, *p* = 0.05; Fig. 4c). As regards vitamin and fatty-acid metabolism, the microbiota of captive sifakas had greater capacity for biosynthesizing phosphopantothenate (PANTO-PWY: log (LDA) = 3.67, *p* = 0.05) and the fatty acids *cis-*vaccenate (PWY-5973: log (LDA) = 4.01, *p* = 0.007) and gondoate (PWY-7663: log (LDA) = 4.00, *p* = 0.008). Concerning plant fiber, sugar, and secondary compound metabolism, captive sifakas harbored microbiota that had greater capacity for D-galactose degradation via the Leloir pathway (PWY-6317: log (LDA) = 3.80, *p* = 0.03; Fig. 4d) and for D-glucuronide and D-glucuronate degradation (GLUCUROCAT-PWY: log (LDA) = 3.46, *p* = 0.03; Fig. 4e).

## Discussion

In a comparative study of gut microbial structure and function in wild versus captive populations, frugo-folivorous lemurs showed both natural, population-level variation and perturbational effects of captivity, but with patterns distinct from other folivorous and herbivorous hosts. The minor differences in microbial community diversity, taxonomic structure, and metabolic pathways among wild sifakas likely reflected environmental heterogeneity across temporal or geographic scales. By comparison, the consortia of captive sifakas had notable signatures of structural imbalance and modest signatures of functional imbalance. Life under human care was associated with greater scores of community diversity, increased signatures of complex fiber, methane, and putative, plant secondary compound metabolism, but decreased signatures of simple fiber metabolism and essential amino-acid biosynthesis. Unusually, these results suggest that these folivores consume a more fibrous and less digestible diet in captivity than in the wild.

Based on alpha diversity metrics, the gut microbiota of sifakas from wild populations comprise a similar number of taxa varying primarily in relative distribution. For example, all wild sifakas harbored similar proportions of *Bacteroidetes* and *Firmicutes,* but the dominant genera, such as *Bacteroides* and members of the *Prevotellaceae* and *Lachnospiraceae* families, differed. Tradeoffs in these taxa are often associated with dietary differences: *Prevotellaceae* members are generally associated with non-celluloytic fiber [40], *Bacteroides* are generally associated with fats and proteins [41], and *Lachnospiraceae* metabolize an array of complex fibers [42]. Further supporting a dietary mechanism of gut microbial variation, the major foods consumed by wild sifakas seemingly co-vary within the dry season. Although published dietary data span few years in different decades [43, 44], Anjajavy versus Ankarafantsika sifakas may eat more young leaves and flowers and less fruit in the dry season. Thus, even small temporal or regional differences in selected or available resources may underlie potentially meaningful variation in microbial action and host nutrition.

Although gut microbiota varied by social group, we only sampled sifakas once and included few lemurs per group, suggesting that our results also could be partially explained by individual identity. Nevertheless, our finding that social-group membership is reflected in gut microbiome structure recapitulates results from previous studies, both in sifakas [45] and other social mammals [46]. Hypotheses put forth to explain gut microbiota structuring by social group typically implicate behavioral mechanisms (i.e., allogrooming and shared contact) or small-scale environmental heterogeneity linked to seasonality or territoriality [46]. In sifakas, both mechanisms may operate. For example, stronger grooming networks correlate to more homogenous microbiota, whereas environmental exposure, as captured by scent-marking frequency, correlates to greater microbiome diversity [45]. Future studies at both Anjajavy and Ankarafantsika could test for finer-grained microbiome structuring by longitudinally sampling individuals, within multiple groups, in relation to social and environmental variables.

Although vastly different from their wild counterparts, captive sifakas nonetheless shared more microbial taxa with their peers in Ankarafantsika, compared to those in Anjajavy. Most (if not all) of the DLC’s founder sifakas originated in and around Ankarafantsika, between 1960 and 1984 (unpublished DLC records). Today, their descendants at the DLC may still retain some features of consortia of the original founders passed down through generations, as reported for other systems [47] and consistent with the ‘heritability’ or vertical transmission of microbiota [48].

The more diverse gut consortia of captive sifakas (compared to wild sifakas) reflects a greater number of microbial genera derived from a greater number of phylogenetic lineages, that is, ‘uncharacteristic’ microbes present at low abundances and that seemingly replaced abundant *Bacteroidetes* members, most notably from the *Prevotellaceae* family and *Bacteroides* genus. The origin of these microbes could perhaps derive from exposure to North Carolinian environmental conditions or to humans. Because biodiversity typically provides functional breadth and redundancy, reflecting community stability, microbial diversity is generally thought to be ecologically beneficial [49] and is often positively associated with community resilience [50] and host health [51]. Nevertheless, the notion that greater diversity inherently reflects or creates more stable or balanced communities should be interpreted with caution. For captive sifakas, greater microbial diversity may reflect microbiome instability or stochasticity, a finding that might be consistent with the ‘Anna Karenina principle’ [52]: the ‘uncharacteristic’ microbes present at low abundances may be transient, failing to colonize the gut because they are unsuccessful at competing for niche space voided by endemic and functional members.

Alternately, because the depletion of *Bacteroidetes* and *Proteobacteria* in the consortia of captive sifakas was accompanied by enrichment for *Firmicutes* and *Euryarchaeota*, differences in taxonomic composition may be linked to dietary tradeoffs. As regards *Bacteroidetes*, the lack of *Prevotella* and other *Prevotellaceae* (bacteria that specialize on non-cellulolytic fibers [40] and appear to be markers for frugivory in wild lemurs [16, 27]), likely reflect the minimal representation of fruit in the diets of captive sifakas. Concurrent gains in *Alistipes* and other *Rikenellaceae* (bacteria associated with high-fat diets [53, 54]) may reflect the nuts and chow provisioned daily to captive sifakas. As regards *Firmicutes*, greater *Clostridiales* from *Lachnospiraceae* and *Ruminococcaceae* (bacterial families that specialize on complex fibers [40, 42]) may reflect greater consumption of foliage by captive versus wild sifakas, and/or of tough versus tender leaves. The enrichment for archael methanogens in the consortia of captive versus wild sifakas also suggests that captive sifakas may be more reliant than their wild peers on gut microbes for recalcitrant fiber fermentation. Methanogens produce methane by metabolizing the by-products of bacterial fermentation under anoxic conditions [55]. In captive sifakas, the lower abundance of *Proteobacteria*, especially of the *Desulfovibrionaceae* family, may owe to fiber-rich diets [56, 57] and slower gut transit times [58]. If so, this finding could help explain why these hosts are notoriously susceptible to infection with enteric pathogens [59, 60], whereas their wild counterparts are not [61–64].

Although our dataset is small and limited by the current scope of representative genes in online catalogues, we observed differences in several microbial functions that may link to populational differences in host metabolism, homeostasis or diet. The enriched metabolic pathways in captive sifakas for the degradation of galactose and glucuronides are putatively associated with greater dietary foliage. Galactose, a monosaccharide sugar, is a component of hemicellulose and pectin fibers that comprise plant cell walls and is bioavailable to gut microbes [65]. Glucuronides, glycosides available to gut microbes [66], can either be ingested or endogenously produced to detoxify xenobiotics and plant secondary compounds [66, 67]. For example, plant polyphenols endogenously converted to glucuronides can re-enter the intestinal tract via the biliary route, where they are metabolized by bacterial *β-*glucuronidases [67]. In a Malagasy rainforest, folivorous indri consume more foliage than do sympatric frugo-folivorous sifakas [68] and have greater microbial metagenomic capacity for galactose, glucuronide, and plant-secondary compound metabolism compared to sympatric sifakas [69]. Although increased glucuronide degradation via gut microbes could relate to substrates other than plant secondary compounds, such findings across lemur species provide further evidence for the microbial response to, and facilitation of, different dietary repertoires across folivorous hosts. Moreover, the increased capacity for both galactose and glucuronide degradation in captive versus wild sifakas bolsters the argument that captive sifakas consume a more foliage-rich diet.

Across species, the capacity for microbially synthesized amino acids varies by host feeding strategy [2]. Moreover, reducing dietary protein, while boosting dietary carbohydrates, can lead to greater de novo biosynthesis by gut microbiota of essential amino acids, especially valine [70]. That captive sifakas in our study had significantly reduced capacity for valine biosynthesis, compared to their wild peers, might suggest that provisioned diets are richer in protein (including e.g., nuts, beans, and chow). In contrast, wild sifakas foraging on protein-limited diets might rely more on gut microbial synthesis to meet their amino-acid requirements. Although microbial metabolism of fiber and plant secondary compounds in folivores and herbivores has received considerable attention, we have much to learn about their microbiota’s role in protein, nitrogen, and amino-acid cycling [2, 70]. In addition, future work to improve the bioinformatic resolution in available online metagenomic databases for wild animals, and to link specific metabolic pathways to specific microbial members, could enhance our ability to characterize wildlife microbiota across conditions.

Beyond contributing evidence to the perturbational effect of captivity on herbivore and folivore gut microbiota [6, 10, 18], we also show that the directionality and strength of effects relate to host-specific feeding strategies [15]. Herbivores and folivores have specialized diets and, presumably, specialized consortia [69]. Such host specificity should be an important consideration when designing husbandry strategies, given that the gut microbes of dietary specialists, compared to those of generalists, may be less resilient to the broad dietary challenges introduced under captive conditions. Because the gut microbiota of captive sifakas respond to minor changes in dietary and housing conditions [29], continued dietary optimization may help reverse captivity-induced dysbiosis [18]. Comparisons of macro and micronutrient food content could identify the specific nutritional discrepancies between the diets of wild and captive conspecifics and inform interventions to test if restoring a more natural or balanced diet (which in sifakas would comprise seasonal fibrous fruits, flowers, more easily digestible vegetables, and immature foliage) would restore more natural and balanced gut microbiota. If effective, these dietary changes could be incorporated into husbandry strategies for captive sifakas and other species facing similar challenges. This study thus provides key mechanistic insight into how feeding ecology and microbiome activity associate with host nutrition and health.

## Conclusions

By providing tools that allow us to probe, monitor, and optimize the nutrition of diverse hosts, microbiome science is poised to offer solutions to enduring challenges in animal husbandry and conservation [4–7, 10, 18]. For wild animals maintained under human care, a current aim is to develop mechanistic understanding of, and methods to reverse, the perturbational effect of captivity on gut microbiota [6, 8–10, 15–18]. Drawing from this expanding body of work, we demonstrate that the perturbational effects of captivity on sifaka gut microbiota are atypical, even opposite those reported in other herbivorous and folivorous hosts [10, 15, 17]. These results highlight the distinction between folivory and frugo-folivory: The nature of captivity-induced dysbiosis likely depends on species-specific dietary discrepancies between foraged and provisioned diets and is not necessarily generalizable across hosts with similar feeding strategies. We argue that greater provisioning of foliage may not be sufficient to restore a balanced microbiota across folivorous species. We also caution against automatically equating microbiome diversity with a more wild-like consortium. Future efforts to alleviate captivity-induced dysbiosis and restore host-microbial symbiosis may require species-specific approaches to naturalizing provisioned diets. Our results across wild sifaka populations show that gut microbiota of folivores are strongly tuned to local conditions [27] and are as specialized as are their hosts [69]. Thus, the extreme sensitivity of folivorous specialists, both to captivity [59, 60, 71] and to anthropogenic change [72], may owe, in part, to co-specialization between hosts and their gut microbes, neither of which can readily adapt to dietary change. Ultimately, we echo the call for microbiome research to be fully incorporated in conservation biology and wildlife management programs of endangered species.

## Methods

### Study subjects and sites

The subjects were 43 adult and 7 subadult Coquerel’s sifakas (Table 1). They included 22 wild sifakas in eight social groups living in the Anjajavy Protected Area, nine wild sifakas in five social groups living in the Ankarafantsika National Park, and 19 semi-free-ranging sifakas in nine social groups maintained at the Duke Lemur Center (DLC). All of the sifakas appeared healthy and were recognizable via distinctive facial or body markings; individuals in the DLC population additionally bore unique collars or tail shaves.

The coastal Anjajavy and inland Ankarafantsika sites are located in northwest Madagascar, 140 km apart and separated by the Mahajamba river system (Fig. 1). Anjajavy boasts 10,803 ha of protected land, 1030 ha (10%) of which form a private reserve near an eco-tourist lodge. It is dominated by forests (including dry deciduous, limestone or ‘tsingy,’ and mangrove forests), interspersed with abandoned agricultural land in various stages of recovery. Ankarafantsika comprises 135,000 ha of dry deciduous forest and various sections of scrub land. Both sites have established trail systems near tourist areas, frequented by large, well-habituated populations of Coquerel’s sifakas.

The DLC, in Durham, North Carolina, maintains the largest breeding population of Coquerel’s sifakas outside of Madagascar. It houses sifakas socially, as mixed-sex pairs or small family groups, in indoor/outdoor stalls, year-round. When ambient temperatures reliably remain above 5 °C, most sifakas gain additional access to large, forested enclosures (0.4–6 ha), in which they can semi-free range and forage freely. The animals receive a daily diet of folivore chow, assorted vegetables, leafy greens, nuts or beans, and local foliage. Water is always freely available.

### Sample collection

We collected one fresh fecal sample per subject (i.e., upon voiding) for amplicon and metagenomic sequencing during week-long missions in Madagascar, conducted from mid-July to mid-August in 2017 and 2018. The samples were collected in the mornings or early afternoons, placed in storage buffer (OMNIgene.GUT, DNA Genotek, Ottawa, Canada) in sterile tubes, and kept out of direct sunlight at ambient temperatures. They were transported at room temperature to Duke University within 2–6 weeks and stored at − 80 °C until analysis.

At the DLC, over a 10-day period in August 2019, we collected one fresh sample per 15 subjects for amplicon sequencing. These samples were split into two aliquots, with one set being preserved in storage buffer as described above, and the other set being placed immediately in sterile tubes on ice and frozen at − 80 °C within 2 h of collection. Both sets of aliquots were used for amplicon sequencing, as described below, and served to test if our sampling methods accurately preserved microbiome composition (see supplementary material). For metagenomic sequencing, we chose high-quality samples from four additional DLC sifakas; these samples had been collected in midsummer 2015 [29] and had been stored only at ultra-cold temperatures.

### Sample extraction and amplicon sequencing

We extracted genomic DNA from all of the fecal samples using commercial kits (QIAGEN DNeasy Powersoil Kit, Hilden Germany) following established protocols [73]. We shipped extracted DNA to the Argonne National Laboratory (Lemont, IL) for sequencing of the V4 region of the 16S rRNA gene, targeting 150 × 150 bp paired-end reads, using the 515f-806r primers and Illumina MiSeq Platform. Using this protocol, we generated 36,613–127,384 reads per sample.

### Amplicon sequencing bioinformatics and statistics

We processed sequence data using an established bioinformatics pipeline in the Quantitative Insights Into Microbial Ecology 2 (QIIME 2) software (version 2019.4) [69, 74]. In brief, sequences were imported into the QIIME environment, demultiplexed, and denoised using DADA2 and default parameters. This process joins paired-end reads, filters out low-quality, singleton, and chimeric reads, and bins sequences into Amplicon Sequence Variants (ASVs) based on 100% sequence identity. Following quality filtering, we removed one ‘Anjajavy’ sample with low read coverage (< 10,000 reads): All remaining samples (or aliquots) were represented by minimally 31,829 useable reads and were retained in downstream analyses.

We assigned taxonomy to ASVs using a de novo trained naïve Bayes classifier built from reads extracted for the 515–806 region from the SILVA 132 database [75]. We removed chloroplast and mitochondrial sequences and created two subsets of the data, one for all samples preserved in buffer from the three study populations and the other for paired aliquots of the 15 DLC samples split between buffer and frozen storage. From each subset, we removed sequences present in only one sample or aliquot and calculated alpha and beta diversity metrics while rarefying to 25,000 sequences/sample. We used Observed ASVs, Shannon’s H index, and Faith’s Phylogenetic Diversity as metrics of alpha diversity, which respectively capture microbiome richness, evenness, and taxonomic representation. We used unweighted and weighted UniFrac distances as our metrics of beta diversity, which respectively capture the proportion of unique taxa between two samples and their relative abundance [76]. Our full bioinformatics pipeline is available online [68].

To determine if wild and captive sifakas had different microbial consortia, we used the first data subset (i.e., samples preserved in storage buffer from all three populations). We tested for differences in alpha diversity, which mostly followed a Gaussian distribution, by performing three analyses of variance (ANOVA) using the aov function in Rstudio (version 0.99.902) [77]; R software program, version 3.3.3 [78]). In each analysis, we used one metric of alpha diversity as the dependent variable, and study population (three categories: Anjajavy, Ankarafantsika, and DLC) and social group nested within population as explanatory variables. We used Tukey’s post-hoc tests to determine significant pairwise comparisons.

Differences in beta diversity were determined by performing permutational multivariate analysis of variance using distance ‘adonis’ analyses and the vegan package (version 2.5–7) [79], in which unweighted or weighted UniFrac distances served as dependent variables, and study population and social group nested within population served as explanatory variables. We used the pairwise.adonis function *post-hoc* to determine the significant pairwise comparisons between study populations. We retained all pairwise comparisons of UniFrac distances for which the sifaka pairs derived from different populations. We performed a Kruskal-Wallis test and used Dunn’s multiple comparison tests post hoc, implemented in GraphPad Prism (version 8.0.2), to determine if the distances between any two sifaka populations differed significantly. Both UniFrac metrics yielded similar findings; we present results of both metrics, but present figures of only unweighted distances (matched figures of weighted distances are available in the supplementary material).

To address taxonomic composition, we determined the microbial genera that were significantly enriched in each of the three populations. Specifically, we collapsed our ASV table at genus-level resolution and performed a Linear Discriminant Analysis Effect Size (LEfSe) [80]. To conservatively account for multiple testing, we used the p.adjust command in R to implement the Benjamini-Hochberg correction factor [81].

For statistical methods and results on the second data subset (i.e., the paired aliquots), see supplementary material.

### Metagenomic sequencing, bioinformatics, and statistics

We analyzed the gut metagenome in a subset of 12 samples, split evenly between the three study populations. For each population, we selected samples from minimally two social groups, targeting samples with high-quality DNA. Those extracts were sent to CosmosID Inc. (Rockville, MD) for library preparation using Illumina’s Nextera XT kit, with a modified protocol [82]. We barcoded, multiplexed, and sequenced the samples on Illumina’s HiSeq X platform, targeting 2 × 150 bp paired-end reads and generated 3.14–13.29 million reads per sample.

The metagenomic sequences were analyzed using a bioinformatics pipeline to determine functional composition. In brief, paired-end reads were trimmed using BBDuk in BBTools (https://sourceforge.net/projects/bbmap/), and only those reads longer than 25 bp were retained. Reads were mapped to the UniRef 90 protein-sequence database using Diamond [83]. Unfortunately, most (91.3–97.2%) of the metagenomic sequences in our samples could not be aligned to online databases, with wild sifakas harboring a significantly greater proportion of unalignable sequences compared to their captive peers (Mann-Whitney test: U = 0, *p* = 0.004). Successful metagenomic read maps were weighted by mapping quality, coverage, and sequence length to estimate gene family abundance [84]. Gene families were annotated to the MetaCyc database to determine the identity and abundance of metabolic pathways per sample [84]. Because our samples varied in sequencing depth, we used Total-sum scaling to normalize the abundance of each pathway to the number of copies per million units. For downstream analyses, we ultimately removed one sample (from Anjajavy) that only mapped to a handful of pathways, as well as all pathways that were present in only one sample. Our final dataset included 11 samples and 142 pathways.

Given the small size of our dataset, we analyzed the relative abundances of retained metabolic pathways relative to whether the sifakas were captive or wild, by collapsing samples from Anjajavy and Ankarafantsika populations. We determined the metabolic pathways that were unique to either wild or captive sifakas. From the subset of pathways that were shared by sifakas across conditions, we used LEfSe to determine those that were differentially enriched in wild versus captive sifakas. We set our *p-*value to 0.10 and did not apply the Benjamini-Hochberg correction factor.

## Supplementary Information

**Additional file 1.**

## Data Availability

Amplicon and metagenomic sequence data are available online (NCBI SRA accession numbers PRJNA495032 & PRJNA684050).

## Abbreviations

DLC: Duke Lemur Center
ASV: Amplicon Sequence Variant
QIIME: Quantitative Insights Into Microbial Ecology
LEfSe: Linear Discriminant Analysis Effect Size
PSC: Plant secondary compound
