# Supplementary material for: Gut microbiota of frugo-folivorous sifakas across environments
Source: Anim Microbiome. 2021 May 18;3:39. doi: 10.1186/s42523-021-00093-5 (PMC8132362; doi:10.1186/s42523-021-00093-5)
Supplement: Supplementary file 1 — Additional file 1. [file 42523_2021_93_MOESM1_ESM.docx]

**Title**: Gut microbiota of frugo-folivorous sifakas across environments

**Authors**: Greene LK, Blanco MB, Rambeloson E, Graubics K, Fanelli B, Colwell RR, & Drea CM

**Supplementary material**

*The effect of storage method on microbiome composition in captive sifakas*

Background and methods: To demonstrate that our storage method accurately preserved microbiota composition, we compared community structure in split fecal samples that were collected from the 15 sifaka subjects housed at the Duke Lemur Center (DLC) and that were stored under two conditions. The first aliquot of each sample was frozen directly at -80 ºC after collection (hereafter the ‘frozen’ condition), a storage method which represents the gold standard in microbiome preservation. The second aliquot of each sample was reported on in the main text: These aliquots were stored in microbiome preservation buffer (OMNIgene.GUT, DNA Genotek, Ottawa, Canada) at ambient temperature for 2 weeks prior to being frozen at -80 ºC (hereafter the ‘buffer’ condition). As this buffer is purported to accurately preserve microbiota composition for up to 8 weeks at ambient temperature [1], we expected to find strong fidelity in microbiome composition between frozen and buffer samples, such that each sifaka’s individual identity and social-group membership would explain more variation in community structure than would storage condition.

We determined microbiome structure in the 15 paired frozen and buffer samples using the amplicon sequencing protocol and QIIME 2 bioinformatic pipeline described in the main text. From generated ASVs, we tested for differences in alpha diversity, using Observed ASVs, the Shannon index, and Faith’s Phylogenetic Diversity, all of which most closely followed the Gaussian distribution. We performed three, paired student’s t-tests using the GraphPad Prism software (version 8.0.2), one per each diversity metric. We tested for differences in beta diversity, specifically the unweighted UniFrac metric, with a permutational multivariate analysis of variance using distance (‘adonis’) implemented in QIIME 2 [2]. We entered unweighted UniFrac distances as the dependent variable, storage condition (buffer vs. frozen), social group, and individual lemur nested within its social group as explanatory variables. We determined if the relative abundance of microbial genera varied with storage condition. Specifically, we collapsed our ASV table at genus-level resolution and performed a Linear Discriminant Analysis Effect Size (LEfSe) [3]. We used the p.adjust command in Rstudio (version 0.99.902) [4] using the R program (version 3.3.3) [5] to adjust for multiple testing using the Benjamini-Hochberg correction factor [6].

Results and discussion: We demonstrate that analyses using frozen versus in-buffer samples yielded similar microbiome composition (figure S1). Regarding alpha diversity, we found small, albeit significant differences between storage conditions for Observed ASVs (t_14_ = 2.497, *p* = 0.026; figure S1a), such that frozen samples had greater diversity compared to buffer samples. Nevertheless, there was no such difference between storage conditions for the Shannon index (t_14_ = 0.832, *p* = 0.419; figure S1b) and only a modest difference between storage conditions for Faith’s Phylogenetic Diversity (t_14_ = 1.847, *p* = 0.086; figure S1c).

Regarding beta diversity, storage condition was significantly associated with microbiome composition, as captured by unweighted UniFrac distances between pairs of samples (PERMANOVA: R^2^ = 0.020, F = 2.526, *p* = 0.006); however, storage condition explained only about 2% of community variation across samples. In contrast, microbiome distances between samples were strongly associated with individual sifaka identity (PERMANOVA: R^2^ = 0.311, F = 4.943, *p* < 0.001) and social-group membership (PERMANOVA: R^2^ = 0.559, F = 11.849, *p* < 0.001), which together accounted for ~87% of the variation across samples (figure S1d).

Paired frozen and buffer samples also had similar taxonomic profiles (figure S1e). LEfSe identified 18 microbial genera with relative abundances that were significantly different between storage conditions; however, following correction for multiple testing, no taxon remained significantly enriched in either frozen or buffer samples.

Ultimately, our two storage conditions yielded nearly identical results and storage condition did not override biologically meaningful patterns in our data.

**Figure S1.** Gut microbiome structure relative to storage condition. Depicted are paired frozen (squares) and buffer (circles) samples. Results of alpha diversity include (a) Observed ASVs, (b) the Shannon index, and (c) Faith’s Phylogenetic Diversity. Results of beta diversity include (d) unweighted UniFrac distances graphed in Principal Coordinate (PCo) space, in which distinct shades represent membership in distinct social groups. Results of community membership include (e) stacked bar charts of the microbial genera that comprised >1% of the total microbiome, in which color families represent microbial phyla and orders, and distinct shades represent microbial genera. ‘Other’ includes the summation of all microbial genera that failed to reach 1% of the total microbiome. * *p* < 0.05; § *p* < 0.1; ns *p* > 0.1.

*Figures and results of weighted UniFrac distances*

In the main text, we presented results of weighted UniFrac distances and here provide the accompanying figures (figure S2). Overall, study population was significantly associated with weighted UniFrac distances (PERMANOVA: R^2^ = 0.71, F_2,25_ = 91.32, *p* < 0.001; figure S2a), which explained more than 71% of the variation across samples. *Post-hoc* pairwise comparisons revealed that sifakas in the two wild populations differed significantly in weighted distances (R^2^ = 0.34, *p* = 0.003); however, the greatest differences in weighted distances were consistently between wild and captive sifakas (R^2^ > 0.93, *p* = 0.003 for both comparisons). Social group, when nested within study population, was significantly associated with weighted UniFrac distances (PERMANOVA: R^2^ = 0.19, F_17,25_ = 2.85, *p* = 0.001), explaining an additional 19% of the variation across samples.

Pairwise comparisons of weighted UniFrac distances between populations further highlight population-level variation. Overall, there were significant differences in mean pairwise distances between the three study populations (Kruskal-Wallis test: H = 387.9, *p* < 0.001), with *post-hoc* tests confirming that the distances between wild-captive pairs, from either wild population, were significantly greater than were the distances between wild-wild pairs (Dunn’s test: both comparisons *p* < 0.001; figure S2b).

**Figure S2.** Weighted UniFrac distances graphed in (a) Principal Coordinate (PCo) space and as (b) pairwise comparisons for Coquerel’s sifakas (*Propithecus coquereli*) living in Madagascar in the Anjajavy Protected Area (up triangle) and Ankarafantsika National Park (down triangle), and in captivity at the Duke Lemur Center (DLC, black circle). *** *p* < 0.001; ns *p* > 0.1.

**References**

1. Brown A, Lynch D, Bouevitch A, Doukhanine E. OMNIgene®• GUT provides easy self-collection and stabilization of liquid fecal samples for microbiome profiling. PD-WP-00056. 2018;1:1-5. www.dnagenotek.com

2. Bolyen E, Rideout JR, Dillon MR, Bokulich NA, Abnet CC, Al-Ghalith GA, et al. Reproducible, interactive, scalable, and extensible microbiome data science using QIIME 2. Nature Biotechnol. 2019;37:852-857.

3. Segata N, Izard J, Waldron L, Gevers D, Miropolsky L, Garrett WS, et al. Metagenomic biomarker discovery and explanation. Genome Biol. 2011;12:R60.

4. RStudio Team. RStudio: Integrated development for R. RStudio. 2020. PBC, Boston, MA URL http://www.rstudio.com/.

5. R Core Team. R: A language and environment for statistical computing. 2017. Vienna, Austria. URL https://www.R-project.org/.

6. Benjamini Y, Hochberg Y. Controlling the false discovery rate: a practical and powerful approach to multiple testing. J R Stat Soc Series B Stat Methodol.1995;1:289-300.
